# Supplementary material for: Decoupling of interacting neuronal populations by time-shifted stimulation through spike-timing-dependent plasticity
Source: PLoS Comput Biol. 2023 Feb 1;19(2):e1010853. doi: 10.1371/journal.pcbi.1010853 (PMC9891531; doi:10.1371/journal.pcbi.1010853)
Supplement: Attachment — Submitted filename: Point-by-Point_Response.pdf [file pcbi.1010853.s006.pdf]

Point-by-point response to reviewers' comments
Manuscript ID: PCOMPBIOL-D-22-01018

Decoupling of interacting neuronal populations by time-shifted stimulation through spike-timing-dependent plasticity

Mojtaba Madadi Asl, Alireza Valizadeh and Peter A. Tass

==

We express our gratitude to the reviewers for their positive evaluation of the manuscript and their suggestions that enabled us to improve the quality and clarity of our study. For your convenience, we highlighted the revised parts in the manuscript in blue.

Data Availability Statement: All relevant data are within the paper and its Supporting Information files. The simulation code is available at https://github.com/MMadadiAsl/Stimulation-induced-decoupling.

==

Reviewer #1:

The authors using model with channels and insert STDP. The authors are able to illustrate that subtle changes to a stimulation protocol can have a significant impact. I believe that the great differential of this work is in the introduction of a time change between the stimuli delivered to two populations of interacting neurons, which can effectively dissociate them. But this should be clearer in the manuscript.

I have only a few indications and later it can be recommended for publication.

We are grateful to the reviewer for the positive judgment of the manuscript. We revised the manuscript in accordance with the reviewer's comments/suggestions.

1) According to the text: the migration of populations was based on initially strong interpopulation links. I suggest that the authors justify this possibility in real human brains.

In accordance with the reviewer’s comment, we discussed increased brain connectivity in the context of several brain disorders in the manuscript.

The following text in the manuscript addresses this point.

Lines 14-27: “Several brain disorders such as Parkinson’s disease (PD) [17-21], essential tremor [22-24], Alzheimer’s disease (AD) [25,26], epilepsy [27-30], schizophrenia [31] and autism spectrum disorder (ASD) [32] are linked with abnormal brain activity and connectivity. For instance, network interactions are severely affected in PD patients due to increased connectivity in cortical/subcortical regions [19,20], that are associated with abnormal neuronal activity [33-35]. Not all neurons and synapses are involved in the pathology to the same extent. For instance, in PD, pallido-subthalamic gamma-aminobutyric acid (GABA)ergic synapses are up-regulated [19], whereas glutamatergic cortico-subthalamic are down-regulated [36]. Abnormal synaptic connectivity may induce abnormally increased neuronal synchrony, as in PD [19,37], or abnormally reduced neuronal synchrony, e.g., in AD [25,26,38], likely due to aberrant synaptic plasticity [37,39-41]. Furthermore, increased long-range brain connectivity may underlie seizure facilitation in patients with epilepsy [30] and may predict symptom severity in ASD [32].”

Lines 81-91: “In the normal brain, information exchange between functionally specialized regions is crucial for higher order brain functions [82,85]. Such an inter-areal communication takes place through long-range synaptic connections across the brain [86-88] and phase synchronization may allow for controlling the inter-areal information exchange by dividing the time into the windows of high and low excitability [85]. Coherence between the activities of brain areas might arise from generic mechanisms across cortex determined by long-range excitatory projections [88]. Abnormal long-range connectivity between brain areas was proposed as a potential pathophysiological mechanism [87,89]. Pathological changes in inter-areal connectivity can cause a functional reorganization of the brain characterized by altered activity and functional connectivity patterns and, thereby impairs the normal brain function [87,89,90].”

References:
Please see the References in the manuscript.

2) Could the authors estimate a final network topology? How? And if possible, what is it for the conditions addressed in this work?

In our model, the two populations were randomly connected to each other via plastic excitatory-to-excitatory synapses. The structural network connectivity (i.e., network topology) does not change in our model and only the strength of these connections is modified by STDP. The final connectivity of the network can be estimated based on the theoretical analysis presented in Fig. 2 and simulation results in Fig. 5 in the manuscript. Fig. 2 roughly predicts the change in the synaptic strengths and, ultimately, the emergent connectivity: Strong bidirectional loops (red region), unidirectional connections (orange regime) and loosely connected neurons (blue regime). Fig. 5 shows how changing stimulation parameters can change the stimulation-induced network activity and connectivity.

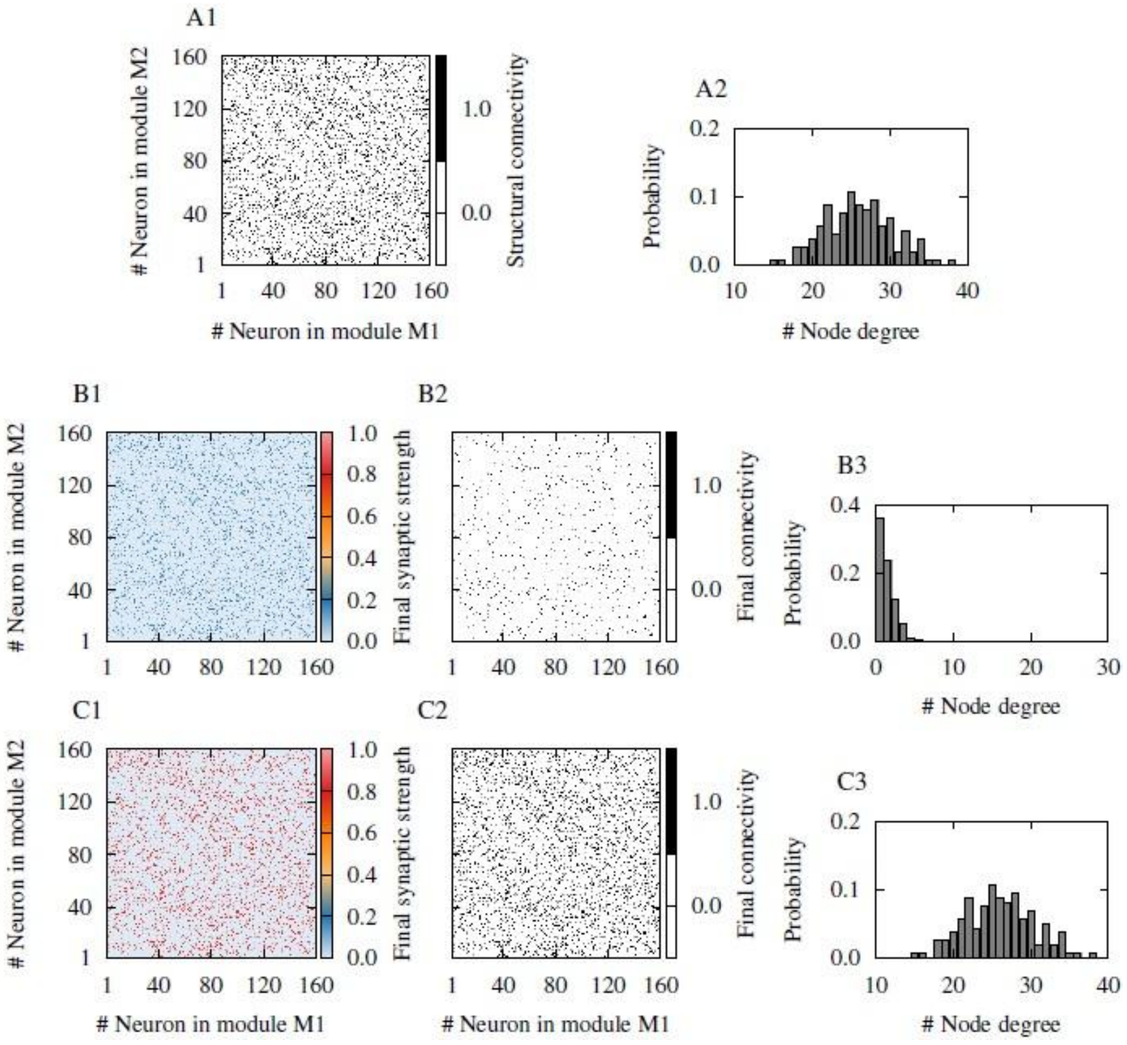

Figure S5. Initial and final network topology. (A1) Structural connectivity matrix of the network (i.e., inter-population excitatory-to-excitatory connections) drawn using a binary representation, i.e., connectivity matrix array, $c_{ij} = 1$ if the two neurons are connected, and $c_{ij} = 0$, otherwise. There were $N_{\text{ex}} = 160$ excitatory neurons within each population. (A2) Degree distribution of the initial network topology with random connection probability $p_{\text{inter}} = 0.15$. (B1) Final synaptic strength matrix of the network. (B2) Binary representation of the final connectivity matrix of the network constructed by introducing a threshold ($h = 0.3$), i.e., connectivity matrix array, $c_{ij} = 1$ if $g_{ij} \geq h$, and $c_{ij} = 0$, otherwise. (B3) Degree distribution of the final network topology. Panels B1-B3 were depicted for the STDP parameters used in Fig. 5B (a) with $(A_+/A_-, \tau_+/\tau_-)$ = (1.6,0.5). (C1-C3) Same as B1-B3, but for the STDP parameters used in Fig. 5B (c) with $(A_+/A_-, \tau_+/\tau_-)$ = (1.6,1.6).

To address this point, we added a new supplementary figure (Fig. S5) and the following text to the manuscript:

Lines 495-511: “The final network topology after the cessation of stimulation can be roughly estimated based on the theoretical results in Fig. 2 and simulation results in Fig. 5, depending on the STDP parameters. The initial structural connectivity matrix of the network is shown in Fig.

S5A1, using a binary representation which follows a random degree distribution (Fig. S5A2). To find the final network topology based on the synaptic strengths (Fig. S5B1), we transformed the structural connectivity matrix into a binary connectivity matrix shown in Fig. S5B2, by introducing a threshold over which the link is maintained in the binary matrix. The final synaptic strength matrix and the final connectivity matrix of the network are shown in Fig. S5B1,B2 and Fig. S5C1,C2 for two different sets of the STDP parameters used in Fig. 5B (a) and Fig. 5B (c), respectively. Particularly, successful stimulation-induced decoupling results in a significantly reduced connectivity (Fig. S5B2) where most of the long-range connections are depressed (Fig. S5B1) so that they are eliminated in the binary connectivity matrix (Fig. S5B3). In contrast, unsuccessful stimulation-induced decoupling is associated with significantly increased synaptic strengths (Fig. S5C1) where the final connectivity is equivalent to the initial structural connectivity (cf. Fig. S5C2 and A1), both following the same random degree distribution (cf. Fig. S5C3 and A2)."

3) Do the authors test the thresholds of synaptic weights so that the collective behavior of the model does not lose its proximity to the realistic situation?

As described related to Eq. (1) in the manuscript (lines 157-171), we used a generic, dimensionless model within which the synaptic weights are scaled and the dynamics can be tuned to mimic those observed in normal and diseased brain in a realistic situation. The synaptic weights are chosen based on the resulting dynamics, i.e., the initial values for the connections are sufficiently strong to result in abnormally strong synchrony and oscillatory dynamics while the final (weak) values lead to low synchrony and irregular activity of the neurons that is a hallmark of healthy brain dynamics, in the context of our model. These dimensionless values can be adequately normalized to conform to the experimentally observed values based on the specific disease and the area under study.

As mentioned in the Methods section (lines 229-235), the synaptic strengths were then updated by an additive rule at each step of the simulation, $g \rightarrow g + \Delta g$. The value of the synaptic strengths was restricted in the range $[g_{\min}, g_{\max}] \in [0.05,1]$. The synaptic strengths were set to $g_{\min}$ ($g_{\max}$) via hard bound saturation constraint once they crossed the lower (upper) bound of their allowed range.

To clarify this point, we added the following text to the manuscript:

Lines 229-231: "We used a generic, dimensionless model within which the synaptic weights are scaled so that they reproduce the dynamics that mimic those observed in normal and diseased brains in a realistic situation."

4) How were the model and conclusions of this work validated?

The model itself is generic and is commonly used as a minimal model for modular complex biological networks [86,99,100]. Accordingly, we here presented a simple and generic stimulation approach that caused pronounced changes of the network dynamics and connectivity, outlasting the cessation of stimulation. The network dynamics/connectivity were tuned to qualitatively mimic the collective and single neuron ones in healthy and diseased brain. To justify our choice of this (established) model, we have discussed this aspect in the manuscript:

Lines 896-914: “The two-neuron motif is a simple, yet well-understood basic model in computational neuroscience [106,109,115,116]. Results of the two-neuron motif served as predictions for the two-population model and were tested by numerical comparison with results obtained in the latter model. On the other hand, the two-population model is an established model for cortical neuronal networks with modular structure [84,86,99,100]. It is widely used to study reliable signal propagation across cortex [84], inter-hemispheric phase coherence despite long-range transmission delays [100], synchronization between oscillations emerging from separated cortical areas [197], as well as in vivo conditioning protocols that produce cortical plasticity [95,96]. In that sense, as demonstrated by our thorough analysis, the comparably simple two-neuron motif represents the dynamics of the two-population model convincingly well. Results obtained in the two-population model provide non-trivial predictions for experimental and clinical tests. The validity of these predictions will, in turn, depend on the validity of the two-population model. While the latter was thoroughly derived [84,86,99,100], it cannot account for all possible additional features of cortical networks, e.g., ranging from layering to glial cells. The success of computational studies [96] to explain experimental outcomes of paired stimulation protocols that produce cortical plasticity [95] suggests that simple cortical models incorporating STDP may be able to explain the neural mechanisms underlying stimulation-induced cortical plasticity between two populations.”

References:
Please see the References in the manuscript.

5) This work can be used to explain cognitive processes? How?

Cognitive processes are related to structure-function interactions [1], in which the product of interconnected brain areas is the basis for higher brain functions such as attention, memory, language, emotion, or executive function. Disruption of normal connectivity and dynamics has been linked to cognitive impairment in a wide range of brain disorders. For example, aberrant increased activity of neurons as well as the pronounced synchronized activity of neuronal populations, and/or pathological synaptic connectivity has been seen in several brain disorders such as Alzheimer’s disease (AD) [2,3], Parkinson’s disease (PD) [4], epilepsy [5], schizophrenia [6] and autism [7]. These changes in the activity and connectivity will affect the communication between the brain areas that is crucial for a variety of the higher order cognitive

functions like memory, perception, attention and motor function [1]. Furthermore, impaired cognitive function in multiple sclerosis (MS) may reflect damage to brain regions due to inflammatory demyelination, thus causing inhibition of axonal transmission. Potentially, impairments of cognitive (and non-cognitive) processes due to abnormal transmission delays have been found MS [8]. Specifically, long-term memory, which is believed to be related to plastic changes of synapses, is one of the most consistently impaired cognitive functions in MS and is seen in 40-65% of patients [9].

Therefore, our study was based on the assumption that the requirement for any treatment of cognitive disorders is to counteract the aberrant activity of the neurons. The aim of our model was to propose a stimulation method to durably reduce abnormal increased activity by unlearning the pathological, increased connectivity. This may ultimately enhance cognitive performance.

To adequately address the referee's question, we added the following text to the manuscript:

Lines 851-862: "Cognitive processes are related to structure-function relationships, in which the product of structurally and functionally interconnected brain areas is the basis for higher brain functions [1]. While we do not claim that our simple models might sufficiently explain complex cognitive processes, it may nevertheless contribute to testable predictions as to how cognitive processes may be impaired, e.g., by abnormal transmission delays. For instance, impaired cognitive function in multiple sclerosis (MS) may reflect damage to brain regions due to inflammatory demyelination, thus causing inhibition of axonal transmission. Potentially, impairments of cognitive (and non-cognitive) processes due to abnormal transmission delays have been found in MS [8]. Specifically, long-term memory, which is believed to be related to plastic changes of synapses, is one of the most consistently impaired cognitive functions in MS and is seen in 40-65% of patients [9]."

References:

[1] Park, H. J., & Friston, K. (2013). Structural and functional brain networks: from connections to cognition. Science, 342(6158), 1238411.

[2] Koelewijn, L., Lancaster, T. M., Linden, D., Dima, D. C., Routley, B. C., Magazzini, L., ... & Singh, K. (2019). Oscillatory hyperactivity and hyperconnectivity in young APOE-ɛ4 carriers and hypoconnectivity in Alzheimer's disease. eLife, 8, e36011.

[3] Schultz, A. P., Chhatwal, J. P., Hedden, T., Mormino, E. C., Hanseeuw, B. J., Sepulcre, J., ... & Sperling, R. A. (2017). Phases of hyperconnectivity and hypoconnectivity in the default mode and salience networks track with amyloid and tau in clinically normal individuals. Journal of Neuroscience, 37(16), 4323-4331.

[4] Fan, K. Y., Baufreton, J., Surmeier, D. J., Chan, C. S., & Bevan, M. D. (2012). Proliferation of external globus pallidus-subthalamic nucleus synapses following degeneration of midbrain dopamine neurons. Journal of Neuroscience, 32(40), 13718-13728.

[5] Vollmar, C., O'Muircheartaigh, J., Barker, G. J., Symms, M. R., Thompson, P., Kumari, V., ... & Koepp, M. J. (2011). Motor system hyperconnectivity in juvenile myoclonic epilepsy: a cognitive functional magnetic resonance imaging study. Brain, 134(6), 1710-1719.
[6] Whitfield-Gabrieli, S., Thermenos, H. W., Milanovic, S., Tsuang, M. T., Faraone, S. V., McCarley, R. W., ... & Seidman, L. J. (2009). Hyperactivity and hyperconnectivity of the default network in schizophrenia and in first-degree relatives of persons with schizophrenia. Proceedings of the National Academy of Sciences, 106(4), 1279-1284.
[7] Supekar, K., Uddin, L. Q., Khouzam, A., Phillips, J., Gaillard, W. D., Kenworthy, L. E., ... & Menon, V. (2013). Brain hyperconnectivity in children with autism and its links to social deficits. Cell reports, 5(3), 738-747.
[8] Chiaravalloti, N. D., & DeLuca, J. (2008). Cognitive impairment in multiple sclerosis. The Lancet Neurology, 7(12), 1139-1151.
[9] Rao, S. M., Grafman, J., DiGiulio, D., Mittenberg, W., Bernardin, L., Leo, G. J., ... & Unverzagt, F. (1993). Memory dysfunction in multiple sclerosis: Its relation to working memory, semantic encoding, and implicit learning. Neuropsychology, 7(3), 364.

6) Recommendations for reading
Motifs in Brains Networks. PLoS Biol. 2004 Nov; 2(11): e369.
Spike timing-dependent plasticity induces non-trivial topology in the Brain. Neural Networks 88 (2017) 58–64.
Effects of the spike timing-dependent plasticity on the synchronization in a random Hodgkin-Huxley neuronal network. Commun Nonlinear Sci Numer Simulat 34 (2016) 12–22.
The human nervous system: Structure and function. https://doi.org/10.1007/978-1-59259-730-7

The suggested papers are now appropriately cited in the manuscript.

===

Reviewer #2:

In this paper, the authors present a computational model of 2 interacting cells/ populations to demonstrate that a) time-shifted stimuli can cause desynchronization and decoupling, and b) that the inclusion of delays into the model shape the model predictions.

It should not be surprising that when the temporal dynamics of the neural units are adjusted so that they violate the LTP conditions of STDP (or conversely, satisfy the conditions of LTD) then the connection strengths between those units are reduced. However, what is novel is the inclusion of realistic delays and some effort to provide predictions that can have immediate utility in clinical settings and I believe that to be the true value of this paper.

What is surprising is that the effect of desynchronization and decoupling persists after the stimulation is removed – and it is this point that is at the core of this model's translational potential and importance.

This paper is extremely well written and is logically structured. One concern is that the model predictions are highly sensitive to the parameters used and not all have been justified or explained (or explored, understandably). I feel that more careful tying of the model to physiological parameters would make this paper stronger.

We thank the reviewer for the positive evaluation of the manuscript and his/her insightful remarks. We revised the manuscript in accordance with the reviewer's comments/suggestions.

Abstract and Introduction

1) There is a slight disconnect in the introduction in that it is focussed almost entirely on PD as an exemplary clinical case where desynchronization of neural circuits has therapeutic potential. However, the model presented is a generalised case that is not 'tuned' to represent any particular region/pathology. I think that the authors should consider ether: a) presenting this as a generalised model and place it in the context of a broader range of pathologies/examples OR b) make the effort to tune the model itself to represent regions implicated in PD specifically.

As the reviewer indicated, we intended to present our results using a generic model. The model suggests a method to suppress the pathologically potentiated long-range connections and the resulting increased activity and hypersynchrony that are observed in a variety of brain disorders. Therefore, we revised the relevant parts of the Introduction section to present our generic model in the context of a broader range of pathologies/examples.

To adequately address the referee's concern, we added the following text to the manuscript:

Lines 14-27: "Several brain disorders such as Parkinson's disease (PD) [17-21], essential tremor [22-24], Alzheimer's disease (AD) [25,26], epilepsy [27-30], schizophrenia [31] and autism spectrum disorder (ASD) [32] are linked with abnormal brain activity and connectivity. For instance, network interactions are severely affected in PD patients due to increased connectivity in cortical/subcortical regions [19,20], that are associated with abnormal neuronal activity [33-35]. Not all neurons and synapses are involved in the pathology to the same extent. For instance, in PD, pallido-subthalamic gamma-aminobutyric acid (GABA)ergic synapses are up-regulated [19], whereas glutamatergic cortico-subthalamic are down-regulated [36]. Abnormal synaptic connectivity may induce abnormally increased neuronal synchrony, as in PD [19,37], or abnormally reduced neuronal synchrony, e.g., in AD [25,26,38], likely due to aberrant synaptic plasticity [37,39-41]. Furthermore, increased long-range brain connectivity may underlie seizure facilitation in patients with epilepsy [30] and may predict symptom severity in ASD [32]."

Lines 28-44: "Therefore, counteracting abnormal changes in brain activity and connectivity by therapeutic interventions may provide effective treatment approaches for brain disorders. Neural circuits in the brain can be modulated by a variety of invasive and noninvasive electrical

stimulation strategies aiming at the recovery of normal circuit functions [42-46]. For instance, noninvasive stimulation of cortical regions can be realized by transcranial direct current stimulation (tDCS) or transcranial alternating current stimulation (tACS) which have shown promising effects on motor and cognitive functions in neuropsychiatric disorders [43,45,47]. On the other hand, invasive high-frequency (> 100 Hz) deep brain stimulation (HF-DBS) is an effective clinical therapy for pathological conditions such as medically refractory PD and epilepsy [42,48,49]. However, reappearance of symptoms soon after the discontinuation of stimulation [50] entails chronic stimulation which may further side effects [51,52]. Also, DBS delivered to the subthalamic nucleus (STN) and globus pallidus internus (GPi) is considered as ineffective for treating impairment of gait and balance and is little beneficial for or even worsens speech impairment [53]. Based on computational studies, it was suggested to counteract abnormal neuronal synchrony by stimulation techniques designed to specifically cause desynchronization [54,55]."

References:
Please see the References in the manuscript.

2) The authors mention the issue of 'pathological connectivity' throughout the paper but I think this could be addressed with more precision. For example, the paper should include a more detailed discussion about the physiological nature of 'increased/ pathological connectivity' or 'strong synaptic connections' in PD (or indeed any other relevant pathologies). Which neuron types are involved/ changes in neural density/ synapses/ receptors/ etc. not to change the model as of course a model is a simplification of the biology, but so that the reader has a better understanding of the physiology in this case and therefore how the model relates.

In accordance with the reviewer's comment, the emergence of long-range, increased brain connectivity in the context of several brain disorders is discussed in the manuscript.

Following text that is added to the revised manuscript is related to the reviewer's comment:

Lines 14-27: "Several brain disorders such as Parkinson's disease (PD) [17-21], essential tremor [22-24], Alzheimer's disease (AD) [25,26], epilepsy [27-30], schizophrenia [31] and autism spectrum disorder (ASD) [32] are linked with abnormal brain activity and connectivity. For instance, network interactions are severely affected in PD patients due to increased connectivity in cortical/subcortical regions [19,20], that are associated with abnormal neuronal activity [33-35]. Not all neurons and synapses are involved in the pathology to the same extent. For instance, in PD, pallido-subthalamic gamma-aminobutyric acid (GABA)ergic synapses are up-regulated [19], whereas glutamatergic cortico-subthalamic are down-regulated [36]. Abnormal synaptic connectivity may induce abnormally increased neuronal synchrony, as in PD [19,37], or abnormally reduced neuronal synchrony, e.g., in AD [25,26,38], likely due to aberrant synaptic

plasticity [37,39-41]. Furthermore, increased long-range brain connectivity may underlie seizure facilitation in patients with epilepsy [30] and may predict symptom severity in ASD [32].”

Lines 81-91: “In the normal brain, information exchange between functionally specialized regions is crucial for higher order brain functions [82,85]. Such an inter-areal communication takes place through long-range synaptic connections across the brain [86-88] and phase synchronization may allow for controlling the inter-areal information exchange by dividing the time into the windows of high and low excitability [85]. Coherence between the activities of brain areas might arise from generic mechanisms across cortex determined by long-range excitatory projections [88]. Abnormal long-range connectivity between brain areas was proposed as a potential pathophysiological mechanism [87,89]. Pathological changes in inter-areal connectivity can cause a functional reorganization of the brain characterized by altered activity and functional connectivity patterns and, thereby impairs the normal brain function [87,89,90].”

References:
Please see the References in the manuscript.

Methods
3) It would be helpful to list the parameters used (with descriptions and references) so it is easy to see what is changed for each simulation. Because the simulations are not listed in methods (which is fine) I had to go back and forth to understand which parameters were changed for each figure and this would be solved with a simple table.

As the reviewer suggested, neuronal, synaptic and network parameters used in simulations are now described/listed in Table 1 in the manuscript in the Methods section. Stimulation parameters including those changed in each figure are listed in Table 2. Plasticity parameters are listed in Table 3 of the revised version.

4) A is used twice as the adjacency matrix and also as a measure of network activity – the authors may wish to change one of these to reduce reader confusion.

The adjacency matrix is now represented by “C” and its corresponding elements by “c”.

5) The temporal parameters of STDP are very fast (10/20 ms) – how have these been determined? I realise there is some exploration of this space in Fig. 5 – but even here these are constrained to be in the ms range which is fast for true LTP/LTD mechanisms.

The time constants of STDP (τ_+ and τ_-) determine the time window for the pairing of the spikes of the pre- and post-synaptic neurons over which the synaptic modification takes place and they were chosen in the range of tens of milliseconds, based on the experimental results [6,7]. But since the change in the synaptic weights per each pair of the spikes is very small (determined by

A_+ and A_-), and due to the noisy nature of the spiking of the neurons that leads to the succession of potentiation and depression, a significant change in the synaptic weights can only be observable after tens of the spike pairs over several minutes [6,7].

To further clarify this point, we added the following text to the manuscript:

Lines 282-291: "We set the parameters of STDP according to the biologically realistic, generic forms of cortical STDP profiles [7] characterized by larger potentiation rate (i.e., $A_+ > A_-$) and relatively longer time window for synaptic depression (i.e., $\tau_+ < \tau_-$). The temporal STDP parameters are consistent with experimental observations, e.g., in rat cortical slices [7]: $\tau_+ =$ 13 ms and $\tau_- = 34$ ms, or those originally reported in rat hippocampal cultures [6]: $\tau_+ = 19$ ms and $\tau_- = 34$ ms. Based on these observations, the direction of the synaptic change critically depends on the relative timing of pre- and postsynaptic spikes on a millisecond time scale [4,5]. Accordingly, STDP time constants of the order of 10-40 ms were widely used in previous STDP modeling studies [1,105,108,109,112]."

References:
Please see the References in the manuscript.

6) What is the external stimulation intended to represent? I agree that modelling it as a simple input current is a sensible first step but there are a few considerations: if it is intended to model tDCS/tACS then this affects E/I cells differently, if it is a sensory stimulus then the authors should justify that it targets E/I cells equally as this is not always thought to be the case (for all stim types).

To clarify this point, we added a new figure (Fig. 7), a new subsection in the Results section and the following text to the revised manuscript, lines "573-600":

Targeting excitatory cells or inhibitory cells during stimulation

"In our model, we assumed the external stimulation as a simple input current separately delivered to the two modules, affecting all excitatory and inhibitory neurons in each population, respectively. To investigate whether the stimulation outcome depends on the type of stimulated cells, i.e., excitatory cells vs. inhibitory cells, we specifically stimulated excitatory neurons in Fig. 5A1 and inhibitory neurons in Fig. 5A2 with the same stimulation and STDP parameters used in Fig. 4 (see Tables 2 and 3). Slightly varied stimulation/model parameters led to similar outcome (not shown). Interestingly, in either case the stimulation outcome is similar to the case where all excitatory and inhibitory neurons were stimulated (Fig. 4). Particularly, the mean synaptic strengths between the two populations were significantly reduced after the cessation of stimulation (Fig. 5B1 and B2). After 5 s of stimulation, both excitatory (Fig. 5C1, top) and inhibitory (Fig. 5C1, bottom) neurons fired at a lower rate when only the excitatory neurons were

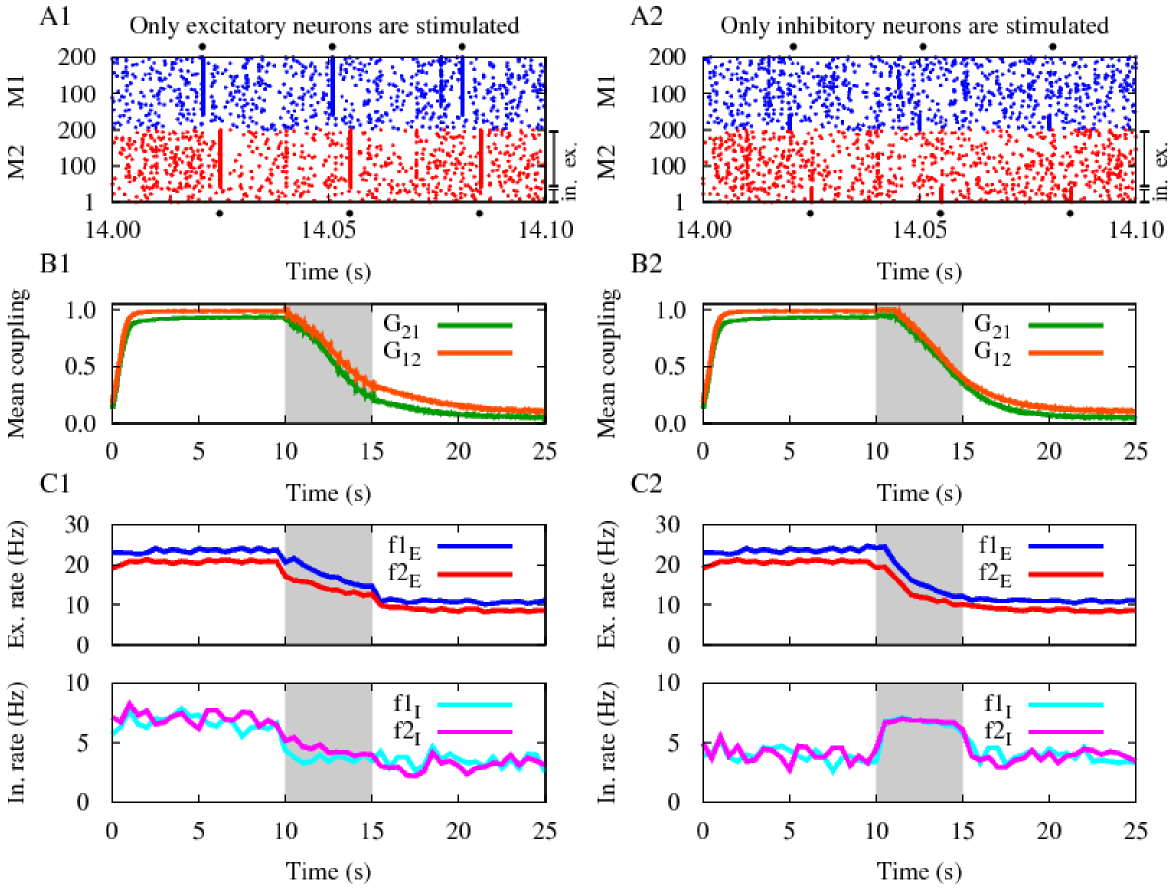

Figure 5. Targeting excitatory cells or inhibitory cells during stimulation. Stimulation outcome when only excitatory neurons (left column) and only inhibitory neurons (right column) were stimulated. (A1,A2) Raster plots are shown for the modules during a 100 ms window of the 5 s stimulation epoch. In each module, neurons #1 to #40 are inhibitory and the remaining are excitatory. For clarity, the onset times of stimulation are indicated with black circles around each panel. (B1,B2) Time course of the inter-population mean coupling. (C1,C2) Time course of the mean firing rates of excitatory (top) and inhibitory (bottom) neurons in each module. Stimulation and STDP parameters were similar to those used in Fig. 4 (see Tables 2 and 3).

stimulated. Following stimulation of only the inhibitory neurons, the firing rate of inhibitory neurons increased during stimulation (Fig. 5C2, bottom), reducing the firing rate of excitatory neurons during and after stimulation (Fig. C2, top).

In a realistic situation, however, depending on whether the external stimulation represents tDCS/tACS or a sensory stimulus, it may affect excitatory and inhibitory cells differentially [132-136]. For instance, inhibitory neurons can be activated at low stimulation intensities, whereas excitatory neurons would require stronger stimulation, as revealed by computational [132-134] as well as experimental findings [135,136]. The stimulation frequency can also differentially modulate the firing rate of excitatory and inhibitory neurons. While at low frequency (i.e., 5 Hz), the firing rate of inhibitory neurons is preferentially increased, at higher frequencies (i.e., 26 and 52 Hz), the excitatory neurons show increased firing rates [133,134],

potentially due to the intrinsic difference in decay times of excitatory and inhibitory synapses [134]. Therefore, careful selection of specific frequencies and amplitudes of the stimulus may allow for selective enhancement and suppression of the excitation-inhibition ratio."

References:
Please see the References in the manuscript.

7) How were the inter-module delays chosen/ which tracts do they represent?

To clarify this point, we added the following text to the manuscript:

Lines 386-395: "Inter-population delays represent the delay in the transmission of signals between the two distant brain regions which interact through long-range projections. Such a range of transmission delays were experimentally observed in cortico-cortical connections, e.g., between primary somatosensory cortex (S1) and secondary somatosensory cortex (S2) in rabbits (~ 2-30 ms) [123] and cats (~ 2-40 ms) [124]. In humans, the average-sized myelinated fiber interconnecting the temporal lobes would have an inter-hemispheric delay of over 25 ms [125,126]. The inter-module delays in our study are chosen to lie within this range of realistic delays for long range connections, but the choices are exemplary (e.g., 5 and 10 ms) and the results can be easily adopted for two specific areas with a known transmission delay."

References:
Please see the References in the manuscript.

8) Do the initial conditions for G/g have any effect on the model predictions?

The initial distribution of the synaptic weights were chosen as strong to give rise to pathological dynamics characterized by overly synchronized neuronal activity of the populations. Model predictions are based on the theoretical results in Fig. 2 and simulation results in Fig. 5 which roughly estimate the emerging stimulation-induced pattern of connectivity. It has been shown previously, that the mean of the initial distribution of the synaptic weights or its standard deviation not only determine the initial dynamics but also the emerging connectivity and dynamics when the connections change through STDP [113,130].

As previously shown in one of our own studies (see Fig. X1 below) [113], in a motif comprised of two neurons and for a given axonal delay (τ_a), the initial values of the synaptic strengths (g_{21}, g_{12}) determine the emergent bi-variate dynamics (blue: in-phase; red: out-of-phase) and connectivity (top left/bottom right corner ($g_{21} \approx 1, g_{12} \approx 0$ or $g_{21} \approx 0, g_{12} \approx 1$): unidirectional connectivity; top right corner ($g_{21} = g_{12} \approx 1$): strong bidirectional connections; bottom left ($g_{21} = g_{12} \approx 0$): decoupled motif).

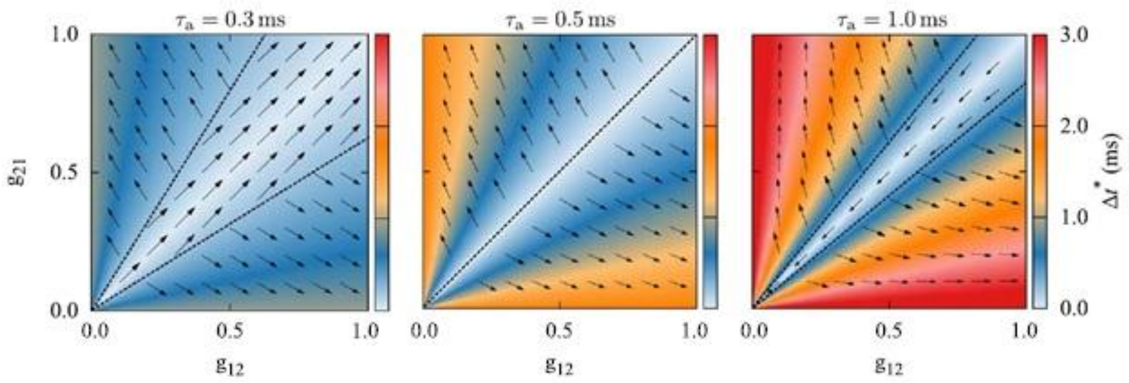

Figure X1. Theoretical prediction of synaptic modification as a function of the initial synaptic strengths. The colors show the stable fixed point of the time lag of spiking of the neurons and the vector field shows the direction of the change of the synaptic strengths. Based on the initial synaptic coupling, different attractors may be achieved: Bidirectional/unidirectional (left column), unidirectional (middle column), and decoupled/unidirectional (right column) state. The dendritic propagation delay was $\tau_d = 0.5$ ms and the axonal delay is denoted above each panel. STDP parameters are $A_+ = A_- = 0.005$ and $\tau_+ = \tau_- = 20$ ms. The figure is taken from Ref. [113].

To address this point, we added the following text to the manuscript:

Lines 448-453: "Moreover, the initial distribution of the synaptic weights was chosen to give rise to pathological dynamics. However, as it has been shown previously, that the mean of the initial distribution of the synaptic weights or its standard deviation could, in principle, affect the emergent dynamics and structure due to multistability of synchronized and desynchronized states [130] or multistability of weak and strong connectivity regimes in plastic neuronal networks [113]."

References:
Please see the References in the manuscript.

9) How is the noisy input determined/ what are the parameters?

This is explained in the revised version of the manuscript:

Lines 172-190: "The noisy input, $I_{\text{ext}}(t)$, represents the external input from other parts of the brain that are not considered in the model, considering the high spiking variability observed in cortex [107], modeled as a homogeneous Poisson process which best represents single neuron dynamics [81,84]. In the model, each excitatory neuron within each module was driven by 8000 independent Poisson excitatory spike trains, each with a mean rate of 1 spike/s. Each inhibitory neuron within each module was driven by 6500 independent Poisson excitatory spike trains, at the same mean rate. The synaptic strengths, transmission delays and external input were tuned such that each population (when isolated) operated in an inhibition-stabilized regime,

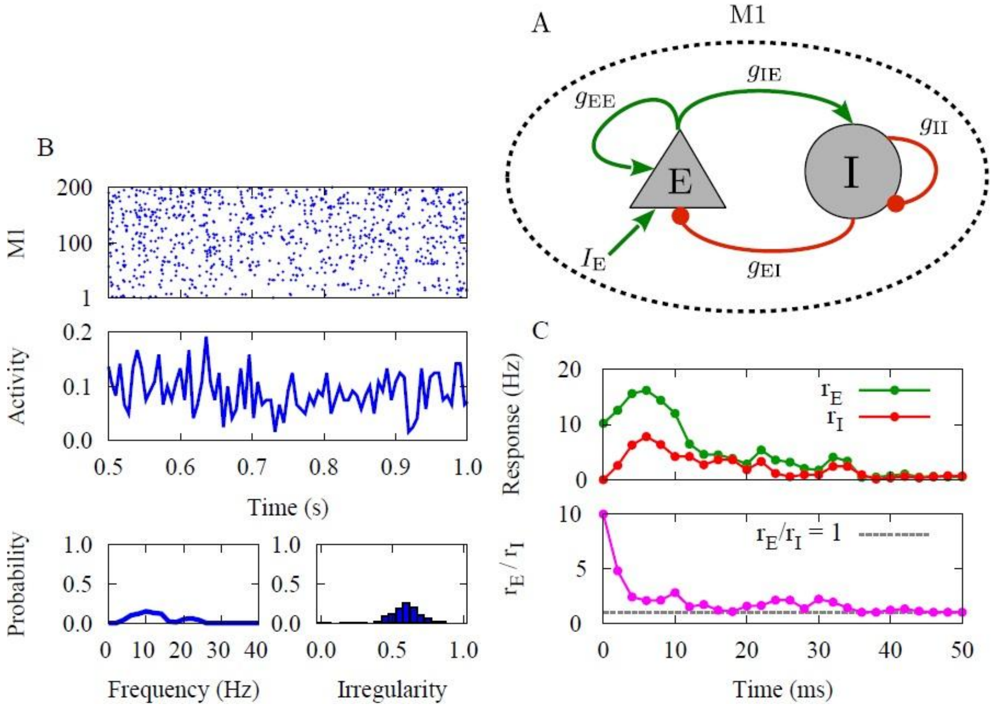

Figure S1. Balanced amplification in the inhibition-stabilized local network. (A) Diagram of the isolated module M1 (with no projections from the module M2) as a balanced circuit with an excitatory (triangle) and an inhibitory (circle) population. Excitatory connections are green and inhibitory connections are red. (B) Raster plot (top) and population activity (middle) of the isolated module M1. (Bottom) Fourier transform frequency of the population activity (left) and distribution of the spike count irregularity (right) of the isolated module M1. (C) (Top) Firing rate of the excitatory (r_E) and inhibitory (r_I) populations in response to a pulse of input (I_E) to the excitatory population at time t = 0 s that sets r_E = 10 Hz (r_I = 0 Hz; representing the baseline of activity). (Bottom) Excitatory-inhibitory response ratio for the top panel.

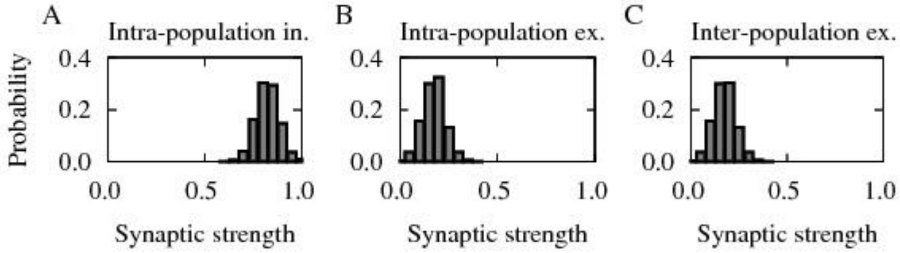

Figure S2. Distribution of the intra- and inter-population synaptic strengths in the model. (A,B) The intra-population inhibitory synaptic strengths were picked from a Gaussian distribution with mean 0.8 and standard deviation 0.05, whereas the intra-population excitatory synaptic strengths were picked from a Gaussian distribution with mean 0.2 and the same standard deviation. (C) The inter-population excitatory synaptic strengths were chosen from a Gaussian distribution with mean 0.2 and standard deviation 0.05.

characterized by irregular individual firing of neurons and desynchronized network activity (see Fig. S1B) [84,102,104]. However, the network operating point was close enough to the oscillatory regime such that strong long-range excitation could push the global activity towards a synchronized regime [84,86,99]. The intra-population inhibitory synaptic strengths were picked from a Gaussian distribution with mean 0.8 and standard deviation 0.05, whereas the intra-population excitatory synaptic strengths were picked from a Gaussian distribution with mean 0.2 and the same standard deviation (see Fig. S2A,B). The inter-population excitatory synaptic strengths were chosen from a Gaussian distribution with mean 0.2 and standard deviation 0.05 (see Fig. S2C)."

References:
Please see the References in the manuscript.

Results
Below I include my interpretation of each result in case I have misunderstood followed by any questions/comments. NB there is some repetition with comments from methods.

10) Fig.2 – pairwise connectivity is shown for the 2-neuron model for a range of delta_t (delays) and T (inter-pulse interval or intra-burst freq). We see that for increasing delays we have more complex patterns of the phase-space (including a desynchronised state for delta_t = 0 and Xi>0).

Correct. We only note that the Δt is the time shift and delay is denoted by ξ. For a discussion on "desynchronized state for $\Delta t = 0$ ms and $\xi > 0$ ms" see below (our response to the point #15).

11) Fig. 3 – time course of the spikes and the connections in 2-neuron model. Firing rates are equivalent for changes in delay indicating this doesn't underlie the changes in synchrony.

The two panels in Fig. 3C were stimulated with different time shifts. According to the results presented in Fig. 2, the time shift of stimulation should be re-tuned when the delay changes. In fact, for the $\xi = 0$ ms delay, a $\Delta t = 15$ ms time shift was used, whereas for the $\xi = 10$ ms delay, time shift was $\Delta t = 5$ ms. This is now explicitly mentioned in Table 2 as well as in the caption of Fig. 3C.

12) Fig. 4 – Raster plots to sow the anti-correlated firing before stim onset within the modules (does the output change if they are correlated?) and then uncorrelated after desynchronization. Bar charts show that correlation decreases while irregularity increases. Spike rate reduces, coupling strength reduces. The persistent effect of the stimulation on desynchronization and decoupling is of great clinical interest. It appears to be driven by lower firing rates post-stimulation. In this case its important to know under what conditions the firing rate remains low after stimulation ceases – for example, the noise level and the local connectivity strengths (E and

I) will play a role in this. A fuller discussion of and justification for the values used would be helpful.

The noise level and local connectivity strengths may actually play a role in this process, however, the synaptic strengths, transmission delays and external input were tuned such that each isolated population operated in an inhibition-stabilized regime, characterized by irregular and uncorrelated firing of neurons (see Fig. S1B) [84,102,104]. However, the network operating point was close enough to the oscillatory regime such that strong long-range excitation could elicit oscillatory global activity [84,86,99]. In fact, initially strong synaptic connections between the two populations led to synchronous firing of the two modules with high firing rate and correlation that is a marker of pathological conditions [18,117], consistent with anatomical evidence that long-range cortical connections are excitatory [118,119]. As shown previously, when this long-range excitation is sufficiently strong, the network dynamics exhibit synchronous oscillations, whereas decoupling of the two populations deteriorates collective oscillations [86,99]. This setting allowed us to study the distinct role of the long-range connections in the emergence of the pathological dynamics, and to inspect the condition for the effective treatment by paired stimulation.

The initial values of the synaptic strengths were chosen based on previous results [113], which roughly predict the emergent STDP-induced connectivity pattern. Our observations revealed that stimulation-induced reduction of the inter-population synaptic strengths below a threshold prevents the re-potentiation of synaptic strengths and, thereby the re-synchronization of the population activity where the firing rates remain low after stimulation cessation. This results in the low-frequency and uncorrelated firing of neurons within each population following stimulation which leads to a depression-dominated modification of the synaptic strengths (according to the STDP profile) and, thereby stabilizes the network's dynamics and connectivity in a loosely connected state.

To further clarify this point, we added the following text to the revised manuscript:

Lines 399-403: "The initial values of the connection strengths were chosen based on the previous results [113]. The neurons in the two populations fired in an irregular and asynchronous manner when the two populations were isolated, while they fire synchronously and the populations oscillated in an anti-phase manner when the two populations were connected by the long-range excitatory projections (Fig. 4A1)."

Lines 420-439: "The persistent effect of the stimulation on desynchronization and decoupling might be of great clinical interest. It appears the persistent effect was driven by lower firing rates and uncorrelated firing of the neurons after the cessation of stimulation. In this case, it is important to know under what conditions the firing rate and the synchrony remains low after

stimulation ceases. The noise level [84] and strength of the local excitatory and inhibitory connections [102,104] may actually play a role in this process; however, we intended to highlight the role of inter-population connections in the emergence and disappearance of the pathologically synchronous activity. To this end, the intra-population synaptic strengths, transmission delays and external input were tuned such that each isolated population worked in a normal, irregular and asynchronous condition where the initially strong synaptic connections between the two populations led to the synchronous firing of the two modules with high firing rate. Our observations in Fig. 5 revealed that stimulation-induced reduction of the inter-population synaptic strengths below a threshold prevents the re-potentiation of synaptic strengths and, thereby the re-synchronization of the population activity where the firing rates remain low after the stimulation cessation. This results in the low-frequency and uncorrelated firing of neurons within each population following stimulation which leads to a depression-dominated modification of the synaptic strengths (according to the STDP profile) and, thereby stabilizes the network's dynamics and connectivity in a loosely connected state."

References:
Please see the References in the manuscript.

13) Fig. 5 – Examines the effects of the STDP parameters on the model behaviour. It seems that the model parameters have a significant effect on the model output. I wonder if the parameters can be tuned to any biological ones so that the reader can get a sense of how these results translate to the brain? I could not see any justification for the choices for tau_+ and tau_- (I assume the learning rates are arbitrary but perhaps these could also be tuned to match empirical data). How have these values been set and are they realistic? LTP /LTD act on longer timescales than this. I think this point is important if these results are to be translated which I assume to be the authors' aim. How is the threshold calculated as it seems to change in each simulation – or is it just an observed value?

Fig. 5 roughly predicts how the imbalance of the STDP parameters in favor of potentiation/depression determines the emerging synaptic structure, e.g., strong bidirectional loop (red region) or a loosely connected structure (blue region) between the populations, as shown in Fig. 5A1,A2. Furthermore, the calculated threshold is just an observed value.

This was earlier explained in response to point #5 raised by reviewer.

The time constants of STDP (τ_+ and τ_-) determine the time window for the pairing of the spikes of the pre- and post-synaptic neurons over which the synaptic modification takes place and they were chosen in the range of tens of milliseconds, based on the experimental results [6,7]. But since the change in the synaptic weights per each pair of the spikes is very small (determined by A_+ and A_-), and due to the noisy nature of the spiking of the neurons that leads to the succession

of potentiation and depression, a significant change in the synaptic weights can only be observable after tens of the spike pairs over several minutes [6,7].

To further clarify this point, we added the following text to the manuscript:

Lines 282-291: “We set the parameters of STDP according to the biologically realistic, generic forms of cortical STDP profiles [7] characterized by larger potentiation rate (i.e., $A_+ > A_-$) and relatively longer time window for synaptic depression (i.e., $\tau_+ < \tau_-$). The temporal STDP parameters are consistent with experimental observations, e.g., in rat cortical slices [7]: $\tau_+ = 13$ ms and $\tau_- = 34$ ms, or those originally reported in rat hippocampal cultures [6]: $\tau_+ = 19$ ms and $\tau_- = 34$ ms. Based on these observations, the direction of the synaptic change critically depends on the relative timing of pre- and postsynaptic spikes on a millisecond time scale [4,5]. Accordingly, STDP time constants of the order of 10-40 ms were widely used in previous STDP modeling studies [1,105,108,109,112].”

References:
Please see the References in the manuscript.

14) Fig. 6 – For Xi fixed (=10) shows the effect of continuous vs burst stim on model predictions and demonstrates that continuous has a more profound effect than burst. This is explained by the fact that there is no STIM-OFF period when the connections start to increase again. In reality, however, the continuous stim would perhaps invoke slower secondary processes such as adaptation which would compensate somewhat for this effect.

We mentioned this in the manuscript, lines 530-533: “In reality, however, the continuous stimulation might invoke slower secondary processes such as adaptation which might counteract the desired effects. However, this remains to be tested experimentally.”

15) A suitable control is demonstrated by using synchronous stimulus. Perhaps I misunderstood but for Xi=10 and delta_t=0, then the model should desynchronise according to Fig. 2? (the parameters would correspond to the point just left of point b in Fig. 2 C). The authors mention jittering at delta_t=0 but this isn’t reflected in Fig. 2.

Thanks for noting this point. This is explained in the in the new version of the manuscript:

Lines 561-572: “Note that the emergent connectivity patterns shown in Fig. 2 are theoretical predictions (see Eq. (5) in Methods) based on deterministic single neuron dynamics evaluated over the time interval between two regular spikes [106]. While the theoretical results roughly predict the stimulation-induced emergent structure of the two-neuron motif and the two populations, the irregular firing of the neurons in the case of two populations makes deviations from the theoretical results. In fact, during stimulation most of the neurons are forced to spike at

the stimulation frequency so that the theoretical predictions are supposed to hold. However, at the extreme points, e.g., at $\Delta t = 0$ as in Fig. 6B2 and B3, the synaptic strengths change only due to the jittering around $\Delta t = 0$ where the potentiation dominates since $A_+ > A_-$. Note that such an effect of jittering which is not reflected in Fig. 2, is only significant for $\Delta t = 0$ and in general for the points lying in the borders of the potentiation/depression areas."

References:
Please see the References in the manuscript.

16) Fig. 7 – For the 2 cases Xi=0, Xi=10 ms then we see the effect of varying delta_t and T (or v) on fano factor and averaged coupling. We get some specific predictions here – for example when Xi=10 delta_t must be between <10 ms and v > 60 Hz or <30 Hz in order to get desynchronization and decoupling. However slight deviations from these parameters produce undesirable effects so prediction precision will be important for translation. This is an important figure.

That is correct. According to the theoretical analysis presented in Fig. 2, the emerging pattern of neuronal activity and synaptic connectivity can be roughly predicted post stimulation based on the time shift (Δt) and inter-pulse interval (T).

We also added the following text to the revised manuscript:

Lines 641-643: "However slight deviations from these parameters produce undesirable effects so prediction precision will be important for translation."

Discussion
Limitations
17) The authors have discussed the limitations of the model satisfactorily.

Great, thank you.

Advance in the field
18) This paper has the potential to advance clinical therapeutic techniques for a range of pathological conditions. In order for this to be the case the authors should try to link the model more closely with the physiology of a specific brain region/regions by justifying the parameters more carefully. It seems clear that the model is highly sensitive to parameter choices and so it's unrealistic to expect direct translation of the model to clinical use but the authors could address next steps in this journey (whole-brain modelling, inclusion of patient specific connectivity patterns, tuning to match EEG spectra, as examples).

To explain this point, we added the following text to the manuscript:

Lines 881-895: “We presented a simple and generic stimulation approach that causes pronounced changes of the network dynamics and connectivity, outlasting the cessation of stimulation. While the conclusions are mainly predictions, the model has the potential to advance clinical therapeutic techniques for a range of pathological conditions. Simple network models can be employed in order to thoroughly understand a wide range of stimulation-induced effects by performing detailed numerical and analytical analysis [72,73,80,130,187-189]. This enables to make predictions that guide the analysis of more detailed and biophysically realistic networks [180,188-193] as well as pre-clinical [74,76,194,195] and clinical studies [75,186,196]. Although variations of model parameters may crucially determine collective dynamics and inter-population connectivity pattern, starting with such simple models and further refining them enables to generate testable predictions for numerical studies in more biophysically realistic models as well as for pre-clinical and clinical studies, e.g., by employing whole-brain modeling and inclusion of patient-specific connectivity patterns tuned to match electrophysiology and neuroimaging data [188,189,193].”

References:
Please see the References in the manuscript.

Discussion of relevant literature

19) There is a good discussion of pathological application of this kind of stimuli. However there is also a body of literature (Dinse, Godde, Vidyasagar) that have looked at the effect of asynchronous inputs to the somatosensory system within a single hemisphere (via digit stimulation) and reported various measurements that can be interpreted as ‘decoupling’ (for example cortical remapping, task performance). I think including these can only strengthen the paper as they are not hypothetical applications, but real ones.

This is now explained in the revised manuscript:

Lines 834-850: “Ultimately, reorganization of cortical networks following stimulation [170-174], e.g., cortical remapping during perceptual performance, can be also interpreted as some sort of decoupling mediated by plasticity [175-177]. For instance, temporally correlated sensory inputs to the digits can lead to the merging of digit representations on the cortical surface, associated with topographic reorganization of the primary somatosensory cortex (SI) evidenced by electroencephalography (EEG), magnetoencephalography (MEG), and functional magnetic resonance imaging (fMRI) [170-174]. Particularly, it was shown that synchronous co-activation of the digits in humans led to an increase in temporal coherence of the fMRI signal due to temporal coincidence between the two-digit inputs, whereas asynchronous co-activation induced no significant change [172,173]. Increased coherency is associated with reduced digit separation for the synchronous input, i.e., cortical representations for synchronously co-activated fingers moved closer together, whereas asynchronously co-activated fingers showed segregated cortical

representations [172,173]. Although mechanisms behind these changes are unclear, stimulation-induced strengthening/weakening of cortical synaptic connections via frequency-dependent synaptic plasticity may be one of the candidates that has been linked to the tactile discrimination performance [175-177].”

References:
Please see the References in the manuscript.

==

Reviewer #3:

In this paper, authors utilized a tractable modeling framework consisting of LIF neurons to theoretically analyze stimulation patterns required to de-synchronize pathological pathways in cortical regions. Although it was not mentioned which brain regions have such potential to be simultaneously stimulated, authors used their methodology to formulate suppression of strong inter-population synaptic connectivity through spike-timing dependent plasticity (STDP). Authors showed that multi-site stimulation parameters can be controlled through their model by incorporating impacts of STDP.

I should clarify that the problem that authors tried to address is very important and timely, and this is why I would like to support this research. A modeling approach is required to tackle such a problem. These two aspects attract my attention. However, I have some big problems with the approach and the problem design that I explain below. Before getting into this point, I should also ask from authors why they have not tried to validate their modeling – probably partially – with some existing neural recordings (like local field potentials) during (or after) deep brain stimulation (although the DBS pulses are delivered to a single population, I think some aspects of the modeling could be validated with experimental data). This is an important point for the development of translational methods. Back to modeling framework, I would like to raise the following points:

We thank the reviewer for the positive evaluation of the manuscript and his insightful comments. We revised the manuscript in accordance with the reviewer's comments/suggestions.

Regarding the reviewer’s suggestion to (partially) validate our modeling: The model itself is generic and is commonly used as a minimal model for modular complex biological networks [86,99,100]. Accordingly, we here presented a simple and generic stimulation approach that caused pronounced changes of the network dynamics and connectivity, outlasting the cessation of stimulation. The network dynamics/connectivity were tuned to qualitatively mimic the collective and single neuron ones in healthy and diseased brain. To justify our choice of this (established) model, we have discussed this aspect in the manuscript:

Lines 896-914: “The two-neuron motif is a simple, yet well-understood basic model in computational neuroscience [106,109,115,116]. Results of the two-neuron motif served as predictions for the two-population model and were tested by numerical comparison with results obtained in the latter model. On the other hand, the two-population model is an established model for cortical neuronal networks with modular structure [84,86,99,100]. It is widely used to study reliable signal propagation across cortex [84], inter-hemispheric phase coherence despite long-range transmission delays [100], synchronization between oscillations emerging from separated cortical areas [197], as well as in vivo conditioning protocols that produce cortical plasticity [95,96]. In that sense, as demonstrated by our thorough analysis, the comparably simple two-neuron motif represents the dynamics of the two-population model convincingly well. Results obtained in the two-population model provide non-trivial predictions for experimental and clinical tests. The validity of these predictions will, in turn, depend on the validity of the two-population model. While the latter was thoroughly derived [84,86,99,100], it cannot account for all possible additional features of cortical networks, e.g., ranging from layering to glial cells. The success of computational studies [96] to explain experimental outcomes of paired stimulation protocols that produce cortical plasticity [95] suggests that simple cortical models incorporating STDP may be able to explain the neural mechanisms underlying stimulation-induced cortical plasticity between two populations.”

References:
Please see the References in the manuscript.

With regard to testing the time-shifted stimulation protocol prior to publishing the theoretical results, e.g., with deep brain stimulation (DBS):

Transparently publishing computationally derived stimulation concepts first in computational/theoretical peer-reviewed journals, thereby openly discussing limitations as well as potential favorable effects, and only later advancing to pre-clinical and clinical tests, was successfully used, e.g., for the development of coordinated reset stimulation, where a number of theoretical papers preceded [1-6] the pre-clinical [7-11] and clinical tests [12-14]. Thereby, the experimental and study protocols were critically informed on relevant aspects from several (preceding) theoretical/computational papers. In fact, based on our experience with IRBs (ethics committees) and our industrial partners, it would be way more difficult (or even impossible) to apply for IRB approval, grant approval and industry cooperation (willingness of manufacturers to modify firmware of implanted pulse generators) to grant permission of clinical tests and establish, e.g., programming tools enabling modified stimulation protocols. In addition, how should we adequately publish, e.g., theory/computational analysis and clinical testing (study protocol and results) in just one paper?

References:

[1] Tass, P. A. (2003). A model of desynchronizing deep brain stimulation with a demand-controlled coordinated reset of neural subpopulations. Biological Cybernetics, 89(2), 81-88.
[2] Tass, P. A., & Majtanik, M. (2006). Long-term anti-kindling effects of desynchronizing brain stimulation: a theoretical study. Biological Cybernetics, 94(1), 58-66.
[3] Hauptmann, C., & Tass, P. A. (2009). Cumulative and after-effects of short and weak coordinated reset stimulation: a modeling study. Journal of Neural Engineering, 6(1), 016004.
[4] Popovych, O. V., & Tass, P. A. (2012). Desynchronizing electrical and sensory coordinated reset neuromodulation. Frontiers in Human Neuroscience, 6, 58.
[5] Manos, T., Zeitler, M., & Tass, P. A. (2018). How stimulation frequency and intensity impact on the long-lasting effects of coordinated reset stimulation. PLoS Computational Biology, 14(5), e1006113.
[6] Kromer, J. A., & Tass, P. A. (2020). Long-lasting desynchronization by decoupling stimulation. Physical Review Research, 2(3), 033101.
[7] Tass, P. A., Silchenko, A. N., Hauptmann, C., Barnikol, U. B., & Speckmann, E. J. (2009). Long-lasting desynchronization in rat hippocampal slice induced by coordinated reset stimulation. Physical Review E, 80(1), 011902.
[8] Tass, P. A., Qin, L., Hauptmann, C., Dovero, S., Bezard, E., Boraud, T., & Meissner, W. G. (2012). Coordinated reset has sustained aftereffects in Parkinsonian monkeys. Annals of Neurology, 72(5), 816-820.
[9] Wang, J., Nebeck, S., Muralidharan, A., Johnson, M. D., Vitek, J. L., & Baker, K. B. (2016). Coordinated reset deep brain stimulation of subthalamic nucleus produces long-lasting, dose-dependent motor improvements in the 1-methyl-4-phenyl-1, 2, 3, 6-tetrahydropyridine non-human primate model of parkinsonism. Brain Stimulation, 9(4), 609-617.
[10] Bore, J. C., Campbell, B. A., Cho, H., Pucci, F., Gopalakrishnan, R., Machado, A. G., & Baker, K. B. (2022). Long-lasting effects of subthalamic nucleus coordinated reset deep brain stimulation in the non-human primate model of parkinsonism: A case report. Brain Stimulation, 15(3), 598-600.
[11] Wang, J., Fergus, S. P., Johnson, L. A., Nebeck, S. D., Zhang, J., Kulkarni, S., ... & Vitek, J. L. (2022). Shuffling Improves the Acute and Carryover Effect of Subthalamic Coordinated Reset Deep Brain Stimulation. Frontiers in Neurology, 13, 716046-716046.
[12] Adamchic, I., Hauptmann, C., Barnikol, U. B., Pawelczyk, N., Popovych, O., Barnikol, T. T., ... & Tass, P. A. (2014). Coordinated reset neuromodulation for Parkinson's disease: proof-of-concept study. Movement Disorders, 29(13), 1679-1684.
[13] Syrkin-Nikolau, J., Neuville, R., O'Day, J., Anidi, C., Koop, M. M., Martin, T., ... & Bronte-Stewart, H. (2018). Coordinated reset vibrotactile stimulation shows prolonged improvement in Parkinson's disease. Movement Disorders, 33(1), 179.
[14] Pfeifer, K. J., Kromer, J. A., Cook, A. J., Hornbeck, T., Lim, E. A., Mortimer, B. J., ... & Tass, P. A. (2021). Coordinated reset vibrotactile stimulation induces sustained cumulative benefits in Parkinson's disease. Frontiers in Physiology, 12, 624317.

1) Although I liked the way the two interacting population of neurons were formulated, why the model is in exact support of authors theoretical hypothesis?, i.e., the oscillations were driven only by synaptic connectivity between two populations and other effects like neuronal activities within each sub-population and background synaptic activities have no effect. 2) What are examples of such neuronal activities/oscillations in some brain regions that authors can provide to support their modeling design?

Of course, effects such as neuronal activities within each sub-population and background synaptic activities play a role in the emergence of oscillations, however, according to previous

studies [84,86,102,104], the model parameters were tuned such that sufficiently strong long-range excitation derives the network dynamics of each EI-module toward synchronous oscillations, whereas decoupling of the two populations deteriorates collective oscillations [84,86,99]. This also means that each isolated EI-module was operating in an inhibition-stabilized regime, characterized by irregular individual firing of neurons (see Fig. S1B) [84,102,104]. However, the network operating point was close enough to the oscillatory regime such that long-range excitation could elicit collective oscillations.

To address the reviewer's concern, we added the following text to the manuscript:

Lines 14-27: "Several brain disorders such as Parkinson's disease (PD) [17-21], essential tremor [22-24], Alzheimer's disease (AD) [25,26], epilepsy [27-30], schizophrenia [31] and autism spectrum disorder (ASD) [32] are linked with abnormal brain activity and connectivity. For instance, network interactions are severely affected in PD patients due to increased connectivity in cortical/subcortical regions [19,20], that are associated with abnormal neuronal activity [33-35]. Not all neurons and synapses are involved in the pathology to the same extent. For instance, in PD, pallido-subthalamic gamma-aminobutyric acid (GABA)ergic synapses are up-regulated [19], whereas glutamatergic cortico-subthalamic are down-regulated [36]. Abnormal synaptic connectivity may induce abnormally increased neuronal synchrony, as in PD [19,37], or abnormally reduced neuronal synchrony, e.g., in AD [25,26,38], likely due to aberrant synaptic plasticity [37,39-41]. Furthermore, increased long-range brain connectivity may underlie seizure facilitation in patients with epilepsy [30] and may predict symptom severity in ASD [32]."

Lines 81-91: "In the normal brain, information exchange between functionally specialized regions is crucial for higher order brain functions [82,85]. Such an inter-areal communication takes place through long-range synaptic connections across the brain [86-88] and phase synchronization may allow for controlling the inter-areal information exchange by dividing the time into the windows of high and low excitability [85]. Coherence between the activities of brain areas might arise from generic mechanisms across cortex determined by long-range excitatory projections [88]. Abnormal long-range connectivity between brain areas was proposed as a potential pathophysiological mechanism [87,89]. Pathological changes in inter-areal connectivity can cause a functional reorganization of the brain characterized by altered activity and functional connectivity patterns and, thereby impairs the normal brain function [87,89,90]."

Lines 172-190: "The noisy input, $I_{\text{ext}}(t)$, represents the external input from other parts of the brain that are not considered in the model, considering the high spiking variability observed in cortex [107], modeled as a homogeneous Poisson process which best represents single neuron dynamics [81,84]. In the model, each excitatory neuron within each module was driven by 8000 independent Poisson excitatory spike trains, each with a mean rate of 1 spike/s. Each inhibitory neuron within each module was driven by 6500 independent Poisson excitatory spike trains, at

the same mean rate. The synaptic strengths, transmission delays and external input were tuned such that each population (when isolated) operated in an inhibition-stabilized regime, characterized by irregular individual firing of neurons and desynchronized network activity (see Fig. S1B) [84,102,104]. However, the network operating point was close enough to the oscillatory regime such that strong long-range excitation could push the global activity towards a synchronized regime [84,86,99]. The intra-population inhibitory synaptic strengths were picked from a Gaussian distribution with mean 0.8 and standard deviation 0.05, whereas the intra-population excitatory synaptic strengths were picked from a Gaussian distribution with mean 0.2 and the same standard deviation (see Fig. S2A,B). The inter-population excitatory synaptic strengths were chosen from a Gaussian distribution with mean 0.2 and standard deviation 0.05 (see Fig. S2C)."

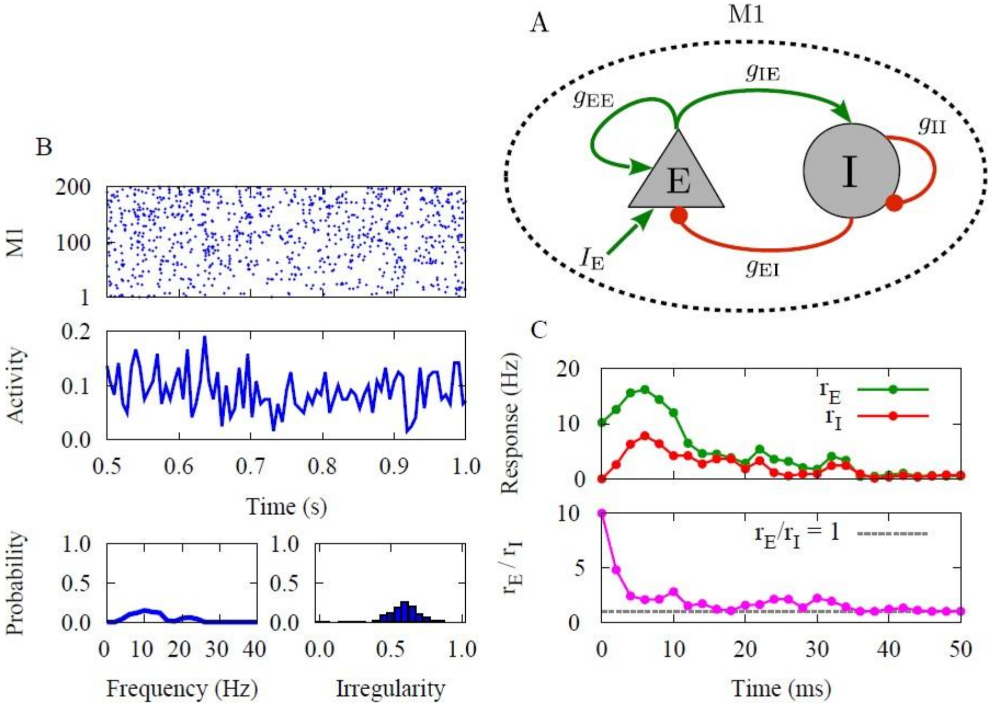

Figure S1. Balanced amplification in the inhibition-stabilized local network. (A) Diagram of the isolated module M1 (with no projections from the module M2) as a balanced circuit with an excitatory (triangle) and an inhibitory (circle) population. Excitatory connections are green and inhibitory connections are red. (B) Raster plot (top) and population activity (middle) of the isolated module M1. (Bottom) Fourier transform frequency of the population activity (left) and distribution of the spike count irregularity (right) of the isolated module M1. (C) (Top) Firing rate of the excitatory (r_E) and inhibitory (r_I) populations in response to a pulse of input (I_E) to the excitatory population at time t = 0 s that sets r_E = 10 Hz (r_I = 0 Hz; representing the baseline of activity). (Bottom) Excitatory-inhibitory response ratio for the top panel.

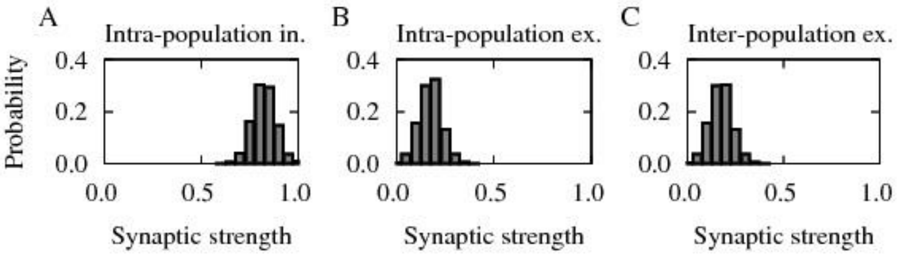

Figure S2. Distribution of the intra- and inter-population synaptic strengths in the model. (A,B) The intra-population inhibitory synaptic strengths were picked from a Gaussian distribution with mean 0.8 and standard deviation 0.05, whereas the intra-population excitatory synaptic strengths were picked from a Gaussian distribution with mean 0.2 and the same standard deviation. (C) The inter-population excitatory synaptic strengths were chosen from a Gaussian distribution with mean 0.2 and standard deviation 0.05.

Lines 351-363: "Weakly coupled excitatory and inhibitory neurons within each population were connected in a sparse manner. The synaptic strengths, transmission delays and external input were tuned such that each population operated in an inhibition-stabilized regime, characterized by irregular individual firing of neurons (see Fig. S1B) [84,102,104]. However, the network operating point was close enough to the oscillatory regime such that strong long-range excitation could elicit pathological synchronous firing [18,117] of the two modules with high firing rate [84,86,99], as consistent with anatomical evidence that cortical excitation has a larger spread than local inhibition due to long-range projections of cortical pyramidal neurons [118,119]. As shown previously, when this long-range excitation is sufficiently strong, the network dynamics exhibit synchronous oscillations, whereas decoupling of the two populations deteriorates collective oscillations [86,99]."

References:
Please see the References in the manuscript.

3) The assumption of two interacting excitatory population, the Hebbian type network, is very simplistic. There are several significant theoretical works in support of inhibitory stabilizing networks and balanced amplification that hugely challenge such simple network modeling. Some works by Kenneth D Miller (as the senior author) in 2009 and 2015 can be considered.

To clarify this point, we added the following text to the manuscript:

Lines 660-678: "In our model, each isolated module is an inhibition-stabilized local balanced network (see Fig. S1A) [102,103] and the two modules interact through long-range excitatory projections [137]. This fundamentally differs from a Hebbian-type network [103], i.e., a single excitatory population of neurons recurrently exciting itself. In our model, each local balanced network consists of both excitatory and inhibitory neurons in 4:1 proportion where the strength of inhibitory synapses within each module is on average 4-fold the strength of excitatory

synapses. Connectivity and network parameters were tuned such that each population operated in an inhibition-stabilized regime, characterized by irregular individual firing of neurons and desynchronized network activity (see Fig. S1B) [102,104]. However, the network operating point was close enough to the oscillatory regime such that strong long-range excitation could elicit oscillatory global activity [84,102,104]. Accordingly, Fig. S1C (top) demonstrates local balanced amplification (LBA) [103,138] in an isolated module in our model which shows the firing rate of the excitatory (r_{E}) and inhibitory (r_{I}) populations in response to a pulse of input (I_{E}) to the excitatory population at time t = 0 s that sets r_{E} = 10 Hz (r_{I} = 0 Hz; representing the baseline of activity), similar to the protocol used by Murphy and Miller (2009) [103]. In this case, increase of the local recurrent excitation is stabilized with stronger inhibition through LBA so that excitation-inhibition ratio is finally balanced (also see Fig. S1C, bottom) [103]."

Lines 151-156: "Each module is a local balanced network (see Fig. S1A) that consists of $N = 200$ LIF neurons randomly connected in a sparse manner [101], of which $N_{\mathrm{ex}} = 0.8N$ were excitatory and $N_{\mathrm{in}} = 0.2N$ were inhibitory in 4:1 proportion. The strength of inhibitory synapses within each module was on average 4-fold the strength of excitatory synapses (see Fig. S2A,B), ensuring local excitation-inhibition balance [102,103]."

Lines 351-363: "Weakly coupled excitatory and inhibitory neurons within each population were connected in a sparse manner. The synaptic strengths, transmission delays and external input were tuned such that each population operated in an inhibition-stabilized regime, characterized by irregular individual firing of neurons (see Fig. S1B) [84,102,104]. However, the network operating point was close enough to the oscillatory regime such that strong long-range excitation could elicit pathological synchronous firing [18,117] of the two modules with high firing rate [84,86,99], as consistent with anatomical evidence that cortical excitation has a larger spread than local inhibition due to long-range projections of cortical pyramidal neurons [118,119]. As shown previously, when this long-range excitation is sufficiently strong, the network dynamics exhibit synchronous oscillations, whereas decoupling of the two populations deteriorates collective oscillations [86,99]."

References:
Please see the References in the manuscript.

4) Why the role of short-term synaptic plasticity was not incorporated, and more importantly, not discussed. Stimulation-induced impact of the short-term synaptic plasticity can have very different effects on glutamatergic and GABAergic synapses, which can affect both inter and intra population interactions.

We focused on stimulation effects that persist after cessation of stimulation which are thought to be mediated by longer lasting models of synaptic plasticity such as STDP and, thereby, we neglected the potential role of short-term synaptic plasticity. While the effects of short-term

synaptic plasticity on model output deserve to be focused on, they add another level of complexity which makes the theoretical analysis and interpretation of results even harder.

As the reviewer suggested, we discussed the potential role of short-term synaptic plasticity in the manuscript:

Lines 771-788: "In this study, we focused on stimulation effects that persist after cessation of stimulation which are thought to be mediated by longer lasting models of synaptic plasticity such as STDP and, thereby, we neglected the potential role of short-term synaptic plasticity. However, stimulation-induced effects of the short-term synaptic plasticity can differently modulate excitatory and inhibitory synapses depending on the frequency as well as the site of stimulation [145-148], which can affect both intra and inter-population interactions. For instance, experimentally it was demonstrated that low-frequency electrical stimulation ($\leq$ 100 Hz) of thalamic structures can induce short-term synaptic facilitation, likely mediated by glutamate release [148,149], whereas stimulation of BG nuclei was associated with short-term synaptic depression, likely mediated by local GABA release [145,146]. This site-specificity may be lost during higher frequencies of electrical stimulation ($>$ 100 Hz) which are usually associated with suppression of neuronal activity due to short-term synaptic depression [148,150,151]. Yet, short-term or long-term synaptic plasticity alone may be insufficient to capture the whole range of stimulation-induced effects in modeling studies [148,151]. Alternatively, incorporation of both short-term and long-term synaptic plasticity in models may provide a more accurate description of synapse-level effects vs. network-level effects of stimulation."

References:
Please see the References in the manuscript.

5) The impact of electrical stimulation was considered with very minimal biophysical constraints. Various studies by McIntyre and colleagues addressed this point.

We are aware of this type of biophysical modeling and its limitations. By the way, in an expert meeting on DBS technology held to finalize the DBS technology review paper [159], a few leading DBS clinicians expressed their significant concerns with the volume of tissue activated (VTA) approach because of the underlying unphysiological assumptions. In any case, as suggested by the reviewer, we added the following text to the revised manuscript:

Lines 789-808: "This paper is meant to serve as conceptual foundation of the time-shifted stimulation of sufficiently distant neuronal populations, applicable with different stimulation modalities. For direct electrical brain stimulation, e.g., DBS, one may additionally calculate the spatial distribution of the electric field to predict the volume of tissue activated (VTA), initially introduced by Rattay (1986) [152]. Of note, this approach considers stimulus effects on a silent axon [153] as opposed to intrinsically active neurons. For instance, VTA-based analysis [154-

156] was used in combination with accurate DBS electrode location [157] and co-registration of patients' brains onto an average template brain [158] in retrospective group-level analyses in large patient cohorts to provide optimal stimulation targets or optimal connectivity patterns for the prediction of favorable outcomes in future patients [159]. However, while the co-registration of VTA and magnetic resonance imaging (MRI) provides a visual approximation, its validity critically depends on the underlying theoretical model, specifically ignoring intrinsic neuronal dynamics and local impedance changes [159]. The same principles and limitations apply to a VTA-based approach for the design of multi-contact leads for multichannel stimulation protocols [160]. Accordingly, more detailed models taking into account representation of the three-dimensional (3D) neuroanatomy, the time-dependent electric field generated by DBS electrodes, and the biophysical mechanisms that regulate the neural response to stimulation [161-164] may shed light on the mechanisms of action of electrical stimulation [165]."

References:
Please see the References in the manuscript.

As a modeler, I totally understand that we cannot expect that a model provides all such details. However, in the absence of experimental data, a theoretical study should address various aspects that a model can offer, rationales for ignoring some constraints, and discussions on the limitation of the model. The question raised in this paper is really an interesting one. I think a more appropriate methodology should be designed.

In accordance with the reviewer's comments, different aspects of the model, rationales behind some assumptions as well as the limitations of the model were carefully addressed (see above). We understand your concerns. Please rest assured that we do not claim that our current study covers all possible aspects. In all modesty, we would like to note that several (expensive) therapeutic neurostimulation trials are based on superficial selections of stimulus parameters and stimulus targets, demonstrating a profound lack of knowledge of any neurostimulation-related advances in the field of computational neuroscience. In contrast, our computational study is meant to be a realistic starting point to bring in computational neuroscience into the clinical arena. Accordingly, we explained the motivation behind our approach in more detail in the manuscript:

Lines 863-880: "Our study is meant to serve as a starting point for a top-down development for time-shifted stimulation protocols administered to remote targets. For comparison, for the improvement of DBS in PD, a top-down approach first used rather simple models, such as phase oscillator networks [72] and later more detailed and advanced models [130,180,181]. Remarkably, already the computational studies in simpler models, e.g., phase oscillator networks, provided key predictions, regarding long-lasting desynchronization [72,131], cumulative desynchronization effects [73] and optimal stimulus patterns [79,182] and parameters [183]. These computational studies revealed non-trivial predications about stimulus-response

characteristics of plastic neuronal networks, specifically the qualitative difference of acute effects (observed during stimulation), acute after-effects (shortly after cessation of stimulation) and long-term after-effects [72,79,140]. In addition, this top-down approach revealed fundamentally different effects of desynchronizing vs. decoupling stimulation patterns [80]. These findings and phenomena were substantially different from what was known about regular DBS. Furthermore, these computationally derived results served as predictions and were critical to the development of appropriate experimental and study protocols, ultimately enabling to verify these predictions in animal experiments [74,76-78,184] and clinical studies [75,185,186].”
